# Induction of hepatic Bach1 mRNA expression by carbon tetrachloride-induced acute liver injury in rats

**DOI:** 10.3892/br.2014.235

**Published:** 2014-02-07

**Authors:** NOHITO TANIOKA, HIROKO SHIMIZU, TORU TAKAHASHI, EMIKO OMORI, KOSUKE KURODA, MARI SHIBATA, MASAKAZU YAMAOKA, YUICHIRO TODA, TAKASHI MATSUSAKI, HIROSHI MORIMATSU

**Affiliations:** 1 Department of Anesthesiology and Resuscitology, Okayama University Medical School, Okayama 700-8558, Japan; 2 Department of Health and Welfare Science, Okayama Prefectural University, Okayama 719-1197, Japan

**Keywords:** Bach1, heme oxygenase-1, free heme, carbon tetrachloride, oxidative stress

## Abstract

Hepatic oxidative stress is a major contributor to the pathogenesis of several acute liver diseases. Diagnostic markers of hepatic oxidative stress may facilitate early detection and intervention. Bach1 is an oxidative stress-responsive transcription factor that represses heme oxygenase 1 (HO-1), the rate-limiting enzyme in the catabolism of heme, a potent pro-oxidant. We previously demonstrated that carbon tetrachloride (CCl_4_) causes oxidative hepatic injury in rats, exacerbated by free heme, suggesting that CCl_4_ may affect Bach1 gene expression. In the present study, we used northern blot analysis to measure Bach1, HO-1 and δ-aminolevulinate synthase (ALAS1; a heme biosynthesis enzyme) mRNA expression levels during acute hepatic injury induced by CCl_4_ (at doses of 0.1, 1.0 and 2.0 ml/kg body weight). Oxidative injury was assessed by measuring serum alanine aminotransferase (ALT), hepatic malondialdehyde (MDA) and glutathione (GSH) content. Treatment with CCl_4_ induced a significant dose-dependent increase in Bach1 mRNA 1–3 h after administration. Bach1 mRNA peaked at 6 h after CCl_4_ treatment (1 ml/kg), followed by a rapid decrease and gradual return to baseline by 12 h after treatment. The timecourse of transient Bach1 mRNA induction roughly mirrored that of HO-1 mRNA, while ALAS1 mRNA was inversely downregulated. Serum ALT levels and hepatic MDA concentration were significantly increased at 24 h after CCl_4_ treatment, while the hepatic GSH content was significantly reduced within 3 h of treatment. Serum ALT levels were positively correlated with Bach1 mRNA levels. These findings indicate that Bach1 mRNA is transiently induced in rat liver by CCl_4_, possibly as a regulatory mechanism to restore HO-1 to baseline following free heme catabolism. Our findings also suggest that Bach1 mRNA expression may be a novel indicator of the extent of oxidative hepatic injury caused by free heme.

## Introduction

Free heme is released through various pathological processes and may further damage tissues by generating reactive oxygen species, such as the hydroxyl radical (1). Heme oxygenase 1 (HO-1) is the rate-limiting enzyme in heme catabolism, thus protecting against heme toxicity. The HO-1 gene (*ho-1*) is induced by free heme and heme-independent oxidative stress and is suppressed by the transcription factor Bach1 (2,3). Under baseline conditions, Bach1 binds to small Maf proteins to form a heterodimer that, in turn, binds to the Maf recognition element (MARE) in the promoter region of *ho-1* to repress transcription (2,3). During oxidative stress and in the presence of excess free heme, Bach1-Maf is released from MARE, allowing transcriptional activation of *ho-1* by nuclear factor (erythroid-derived 2)-like 2 (Nrf2)-Maf heterodimers (2,3).

Carbon tetrachloride (CCl_4_) was shown to cause severe hepatic injury in animals (4,5). CCl_4_ is reductively metabolized by hepatic cytochrome P450 (CYP), producing a reactive intermediate that catalyzes the production of lipid peroxides. This early lipid peroxidation initiates an oxidation cycle that eventually results in the breakdown of cell membranes (4). We previously demonstrated that the treatment of rats with CCl_4_ led to a rapid increase in microsomal heme concentration, which was likely due to the destruction of hepatic CYP and the significant HO-1 induction in hepatocytes (5). The concurrent inhibition of HO-1 activity resulted in a sustained increase in the microsomal heme concentration, aggravation of hepatic injury and exacerbation of the inflammatory response, suggesting that increased free heme plays a significant role in CCl_4_-induced oxidative injury (5).

Considering that Bach1 inactivation and CCl_4_-induced tissue injury are caused by heme-dependent oxidative stress, CCl_4_ may also regulate Bach1 expression. To test this hypothesis, rats were treated with CCl_4_ and the mRNA expression levels of Bach1, HO-1 and δ-aminolevulinate synthase (ALAS1; a heme biosynthesis enzyme), were measured to assess hepatic oxidative injury.

## Materials and methods

### Animals and treatments

A total of 64 male Sprague-Dawley rats weighing 200–260 g were purchased from Clea Japan, Inc. (Tokyo, Japan) and housed in a temperature-controlled room (25°C) with alternating 12-h light/dark cycles and were allowed free access to water and chow until the start of the experiments.

The rats were randomly divided into two groups, the CCl_4_-treated (n=44) and vehicle-treated (control) (n=20) groups. The rats in the CCl_4_ group were intraperitoneally (i.p.) injected with CCl_4_ (Sigma-Aldrich Japan Co., Tokyo, Japan) at doses of 0.1, 1.0 and 2.0 ml/kg body weight, dissolved in an equal volume of corn oil. The control rats were i.p. injected with the same volume of corn oil. Under light ether anesthesia, the rats were sacrificed at each predefined time point (0–24 h) by exsanguinations from the abdominal aorta. Briefly, the abdominal cavity was opened, blood was collected through a catheter inserted into the aorta and the liver was excised. The livers were immediately frozen in liquid nitrogen and stored at −80°C until RNA extraction. For determination of hepatic malondialdehyde (MDA) and glutathione (GSH) content, the livers were perfused *in situ* via the abdominal aorta with ice-cold 0.9% NaCl solution until the venous effluent became clear. The livers were then removed, frozen and stored as described above.

The animal experiments were approved by the Animal Use and Care Committee of Okayama University Medical School (Okayama, Japan). The care and handling of the animals were in accordance with the National Institutes of Health Guidelines for Animal Research.

### cDNA probes

The template cDNAs used to generate probes for northern blot analysis included rat pRHO-1 (6), provided by Dr S. Shibahara, rat pKRA2cA (7) and ALAS1, provided by Dr M. Yamamoto, and rat Bach1 cDNA, corresponding to base pairs 785–1382, provided by Dr K. Igarashi (Tohoku University, Sendai, Japan). The rat Bach1 cDNA was prepared from C6 glioma RNA by reverse transcription and polymerase chain reaction and constructed in a pCR^®^-Blunt II-TOPO^®^ Vector (Invitrogen Life Technologies, Carlsbad, CA, USA) (8). All the cDNA probes used for northern blot analysis were labeled with [α-^32^P]dCTP (PerkinElmer, Inc., Yokohama, Japan) using the Amersham Rediprime II DNA labeling system (GE Healthcare Japan Co., Tokyo, Japan) according to the manufacturer’s instructions.

### RNA isolation and northern blot analysis

Total RNA was isolated from the rat tissues using TRI-Reagent^®^ (Sigma-Aldrich Japan Co.) according to the manufacturer’s instructions. Northern blot analysis was performed as previously described (9). Briefly, total RNA (20 μg) was separated by electrophoresis on 1.2% (w/v) agarose gel containing 6.5% (v/v) formaldehyde. After blotting on a sheet of Bio-Rad Zeta-Probe GT blotting membrane (Bio-Rad Laboratories, Richmond, CA, USA), RNA samples were hybridized with [α-^32^P]dCTP-labeled cDNA probes, followed by washing under stringent conditions. Each blotted membrane was exposed to a sheet of Fuji Medical X-ray film (Fujifilm Corp., Tokyo, Japan) with an intensifying screen at −80°C. Target bands, as well as the 18S ribosomal RNA band on autoradiographs, were quantified using a Gel Print™ 2000i image scanner and Basic Quantifier™ version 3.0 image analysis software (Genomic Solutions, Inc., Ann Arbor, MI, USA). The relative amounts of hybridized radiolabeled cDNAs were normalized to 18S ribosomal RNA levels to correct for differences in gel loading.

### Assay of serum ALT activity

Serum was separated from whole blood by centrifugation at 1,600 × g for 10 min at room temperature and serum ALT activity was measured using an automatic biochemical analyzer calibrated with quality control standards (Dade Dimension^®^ AR^®^ Clinical Chemistry system; Global Medical Instrumentation, Inc., Ramsey, MN, USA).

### Measurement of hepatic MDA concentration

Hepatic tissues were homogenized in 9 volumes of 0.1 M phosphate buffer (pH 7.4) (w/v) containing 5 mM butylated hydroxytoluene (BHT) using a Potter-Elvehjem type glass-Teflin homogenizer (AGC Techno Glass Co., Ltd., Shizuoka, Japan). BHT was provided as a 500-mM solution in acetonitrile. Homogenized liver samples were filtered through mesh gauze and the MDA concentration was measured using the Bioxytech^®^ MDA-586™ kit (Oxis International, Inc., Foster City, CA, USA) according to the manufacturer’s instructions. The results are expressed as μmol MDA/mg protein. The protein concentration in the homogenized liver samples was measured using the DC™ protein assay (Bio-Rad Laboratories, Hercules, CA, USA).

### Measurements of hepatic GSH content

Hepatic tissues were minced in 10 volumes of ice-cold aqueous 5% metaphosphoric acid. The homogenized samples were centrifuged at 3,000 × g for 10 min at 4°C and the upper clear aqueous layer was collected for analysis. The GSH assays were performed using the Bioxytech^®^ GSH-400™ kit (Oxis International, Inc.) according to the manufacturer’s instructions. The GSH content is expressed as μmol/g fresh tissue weight.

### Statistical analysis

Data are presented as the means ± standard deviation. Continuous variables were compared by Student’s t-tests or analysis of variance followed by Tukey-Kramer’s honestly significant difference post hoc tests, as appropriate. The correlation between Bach1 mRNA and serum ALT levels was assessed by Pearson’s correlation coefficient and expressed in r^2^ and P-values. The JMP 10™ package (SAS Institute, Inc., Cary, NC, USA) was used for all the statistical calculations. P<0.05 was considered to indicate a statistically significant difference.

## Results and Discussion

### Effects of CCl_4_ treatment on serum ALT, MDA and GSH levels

Serum ALT activity was assessed 24 h after i.p. injection of CCl_4_ (1 ml/kg) as a measure of hepatic dysfunction. The serum ALT levels in the CCl_4_-treated rats were significantly increased compared to those in the control rats (Table I). In this model, hepatic injury is considered to be caused by CCl_4_-mediated free radical production and ensuing lipid peroxidation (4); thus, we determined hepatic MDA levels 24 h after CCl_4_ treatment. Hepatic tissue samples from the CCl_4_-treated rats exhibited significantly elevated MDA levels compared to the samples from the vehicle-treated controls (Table I) (5). Consistent with CCl_4_-induced oxidative stress (10), hepatic tissue homogenates from the CCl_4_-treated rats exhibited a significantly lower GSH content compared to samples from the vehicle-treated controls, reaching a nadir of ~75% of the baseline at 3 h after injection (Table I).

### Effects of CCl_4_ treatment on HO-1 and ALAS1 gene expression

**Figure 1:**
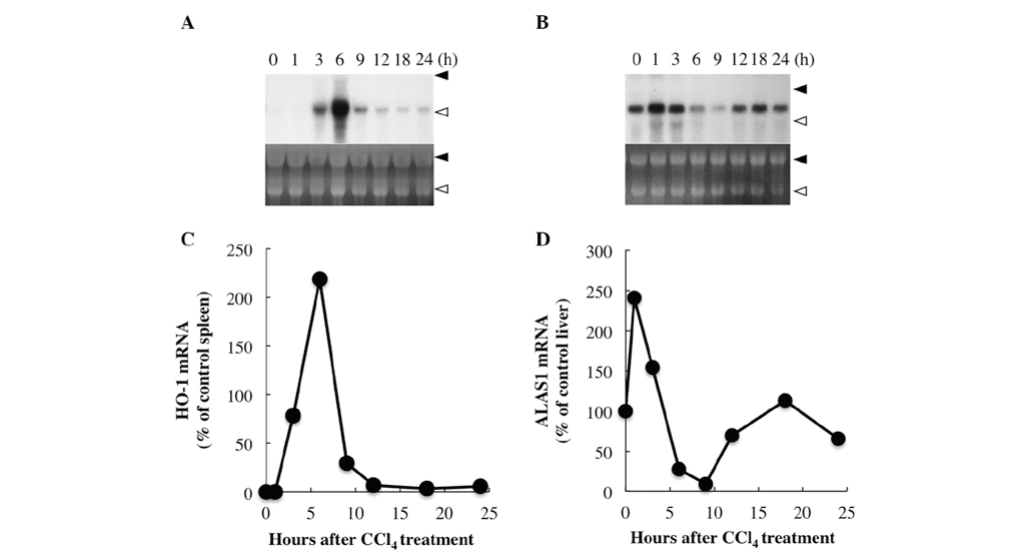
Changes in hepatic HO-1 and ALAS1 gene expression levels following CCl_4_ treatment. The rats were sacrificed at 0, 1, 3, 6, 9, 12, 18 and 24 h after injection of CCl_4_ (1.0 ml/kg, intraperitoneally), their livers were excised and total RNA (20 μg) was subjected to northern blot analysis. Autoradiographic signals of RNA blots hybridized with (A) [α-^32^P]dCTP-labeled HO-1 or (B) ALAS1 cDNA probes are shown. Ethidium bromide staining of the same RNA is shown as the loading control. Closed arrowhead, 28S ribosomal RNA; and open arrowhead, 18S ribosomal RNA. Three independent experiments showed similar results and a typical example is shown. (C) Concentrations of HO-1 mRNA are expressed as relative values to the concentration of an untreated control spleen, in which HO-1 is known to be constitutively expressed; and (D) concentrations of ALAS1 mRNA are expressed as relative values to the concentration of an untreated control liver. HO-1, heme oxygenase-1; ALAS1, δ-aminolevulinate synthase; CCl_4_, carbon tetrachloride.

It was previously demonstrated that CCl_4_ treatment increases the microsomal free heme concentration, which may exert marked effects on the heme regulatory enzymes ALAS1 (biosynthesis) and HO-1 (catabolism) (11,12). HO-1 mRNA expression was barely detectable in the vehicle-treated control liver (Fig. 1A); however, it started to increase 1–3 h after CCl_4_ injection, reaching a maximum at 6 h prior to a rapid decrease and a gradual return to near baseline levels by 12 h (Fig. 1A). Unlike HO-1 expression, the levels of hepatic ALAS1 mRNA, which is the target of heme feedback control (7), increased immediately on CCl_4_ injection, but decreased below baseline by 6 h after treatment (at the peak time of HO-1 mRNA expression). Hepatic ALAS1 mRNA expression reached a minimum of ~10% of that in the untreated control liver at ~9 h after treatment, followed by a gradual increase and return to baseline by ~18 h after treatment (Fig. 1B). These changes are consistent with those reported by our previous study (5). HO-1 was found to be upregulated, whereas ALAS1 was downregulated by heme (7,12). Thus, the reciprocal responses of the HO-1 and ALAS1 genes strongly suggest an increase in hepatic intracellular free heme (13) following CCl_4_ treatment. We previously demonstrated that inhibition of HO-1 resulted in a sustained increase in the hepatic free heme concentration, possibly due to CCl_4_-mediated destruction of hepatic CYP and exacerbation of CCl_4_-induced hepatic injury (5). Thus, during CCl_4_-induced hepatic injury, it is reasonable to hypothesize an increase in intracellular heme concentration, which may compound free radical production and exacerbate cell oxidative injury.

### Effects of CCl_4_ treatment on Bach1 gene expression

**Figure 2:**
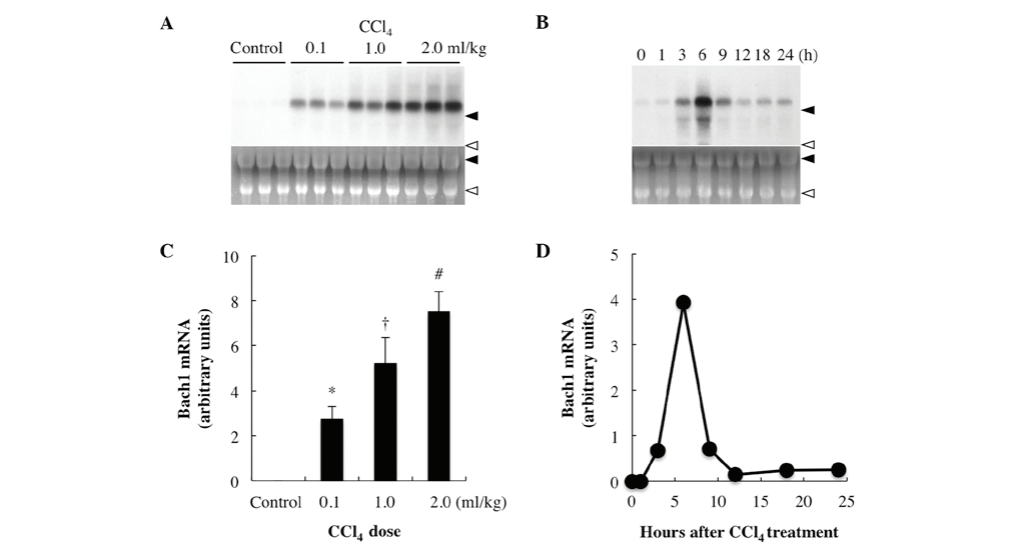
Effect of CCl_4_ treatment on Bach1 gene expression. (A) Dose-response. The rats were injected with either CCl_4_ [0.1, 1.0 or 2.0 ml/kg body weight, intraperitoneally (i.p.)] or 2 ml of vehicle (corn oil), were sacrificed 6 h after injection and their livers were excised for northern blot analysis. (B) Timecourse of hepatic Bach1 gene expression after CCl_4_ treatment. The rats were sacrificed at 0, 1, 3, 6, 9, 12, 18 and 24 h after CCl_4_ injection (1.0 ml/kg, i.p.) and their livers were excised. Total RNA (20 μg) was subjected to northern blot analysis. Autoradiographic signals of RNA blots hybridized with a [α-^32^P]dCTP-labeled Bach1 cDNA probe are shown. Ethidium bromide staining of the same RNA is shown as the loading control. Closed arrowhead, 28S ribosomal RNA; and open arrowhead, 18S ribosomal RNA; control, vehicle (corn oil)-treated rats. Three independent experiments yielded similar results and a typical example is shown. (C and D) Bach1 gene expression levels expressed as densitometric arbitrary units. Data are expressed as the means ± standard deviation and were statistically evaluated using analysis of variance followed by Tukey-Kramer’s honestly significant difference test. ^*^P<0.05 vs. control group; ^†^P<0.05 vs. 0.1 ml/kg CCl_4_; and ^#^P<0.05 vs. 1.0 ml/kg CCl_4_. CCl_4_, carbon tetrachloride.

The Bach1 transcription factor acts as a repressor of *ho-1* activation (2,3). Under physiological conditions, Bach1 forms heterodimers with the basic leucine zipper subfamily of small Maf proteins that bind to the MARE in the promoter region of *ho-1* and repress transcription (2,3). During oxidative stress, Bach1 is released from MARE, allowing transcriptional activation of *ho-1* by Nrf2-Maf heterodimers (2,3). An increase in the intracellular heme concentration appears to release Bach1 from MARE and promote Bach1 nuclear export by directly binding to heme-binding motifs (Bach1 CP motifs), which in turn allows the transcriptional activation of *ho-1* (2,3). As previously mentioned, CCl_4_ treatment induces hepatic oxidative damage that is dependent, at least in part, on free heme accumulation. Thus, CCl_4_ treatment may also affect hepatic Bach1 expression. While Bach1 mRNA was not detectable in the livers of the vehicle-treated control rats, its expression was significantly increased in the livers of the rats injected with ≥0.5 ml/kg CCl_4_ and the increase was dose-dependent (≤2.0 ml/kg) (Fig. 2A). Following treatment with 1 ml/kg CCl_4_, hepatic Bach1 mRNA expression started to increase after 1–3 h, reaching a maximum at 6 h prior to returning to near baseline levels by 12 h (Fig. 2B).

To the best of our knowledge, this is the first study to demonstrate the induction of Bach1 mRNA *in vivo*. Consistent with our findings, a recent cell culture study reported that Bach1 mRNA was upregulated by oxidative stress evoked by ultraviolet A irradiation, a treatment that releases free heme from microsomal heme-containing proteins (14,15). Hypoxia, desferrioxamine and interferon-γ are among the other treatments known to upregulate Bach1 in cultured cells (8). However, the elevated expression of Bach1 repressed HO-1 expression in human vascular endothelial, T98G glioblastoma and A549 lung cancer cells (8). Furthermore, interleukin-γ decreased HO-1 expression through Bach1 induction in human retinal pigment epithelial cells. By contrast, hypoxia induced HO-1 and Bach1 mRNA expression (8). Thus, the association between Bach1 and HO-1 mRNA expression appears to differ according to the stimulus and may also be cell-specific. Therefore, individual hepatic cell types may respond differently to CCl_4_ treatment. In this case, the observed Bach1 expression response reflects all the cell types according to response strength and cell fraction.

**Figure 3:**
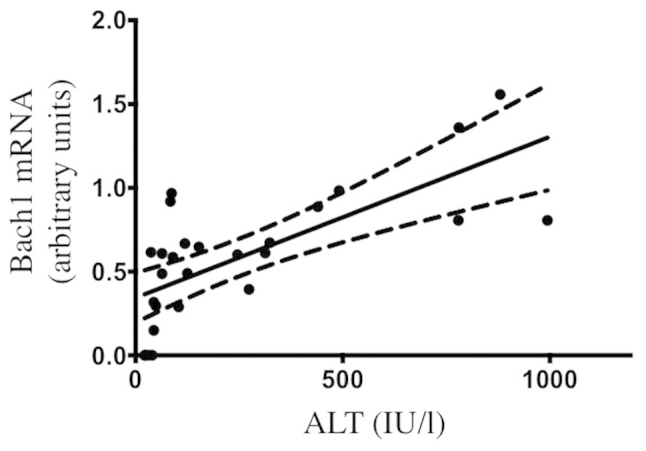
Correlation between hepatic Bach1 gene expression and serum ALT levels following CCl_4_ treatment. The rats were injected with CCl_4_ (0.1, 1.0 or 2.0 ml/kg body weight, intraperitoneally) or vehicle (corn oil) and were sacrificed after 6 h. The livers were excised for northern blot analysis and whole blood was collected for examination of serum ALT activity 6 h after CCl_4_ injection. A significantly positive correlation between Bach1 gene expression and serum ALT levels was observed (r^2^=0.503342; P<0.0001). Linear regression (solid line) with 95% confidence intervals (dotted lines) is presented. ALT, alanine aminotransferase; CCl_4_, carbon tetrachloride.

Although heme proteins are necessary for cell viability, excess free heme is deleterious, as it acts as a potent pro-oxidant (16). In fact, failure to control the deleterious effects of free heme contributes to the pathogenesis of a number of conditions, such as severe sepsis, malaria and hemolysis associated with large-volume transfusion (16), underscoring the utility of the CCl_4_-induced hepatic injury model (5). As illustrated in Fig. 3, the serum ALT levels at 6 h after treatment were positively correlated with Bach1 mRNA levels (r^2^=0.5033, P<0.001). Taken together, our findings suggest that Bach1 mRNA expression may reflect the extent of oxidative tissue injury aggravated by free heme.

The pathophysiological significance of Bach1 mRNA induction by oxidative injury remains to be determined. The activation of HO-1 by CCl_4_ is a compensatory stress response that results in the clearance of excess heme (5). However, the overexpression of HO-1 is likely to have deleterious consequences once this excess free heme is catabolized, as HO-1 itself damages heme-containing proteins and releases labile iron, which also catalyzes free radical formation (17). Thus, the activation of Bach1 expression by CCl_4_ may be a crucial compensatory mechanism to accelerate the restoration of homeostasis under this unique form of oxidative stress (15). Further studies measuring free heme, Bach1 protein expression and subcellular localization and HO-1 expression in tandem are required to test this hypothesis.

In conclusion, to the best of our knowledge, this study was the first to demonstrate that Bach1 mRNA is induced in rat liver following i.p. administration of CCl_4_, a compound that causes hepatic oxidative injury mediated partly through increasing the concentration of free heme. A significant positive correlation between hepatic Bach1 gene expression and serum ALT levels following CCl_4_ treatment was also demonstrated, suggesting that Bach1 mRNA expression may reflect the extent of CCl_4_-induced oxidative tissue injury.

## Figures and Tables

**Table I:** Effects of CCl_4_ treatment on serum ALT, hepatic MDA and GSH concentrations.

	Experimental groups		
		
Measurements	Control	CCl_4_	P-values
ALT (IU/l) (n=8)	32.13±3.68	384.38±333.39	0.05
MDA (μmol/mg protein) (n=6)	0.19±0.01	0.29±0.04	0.005
GSH (μmol/g FW) (n=6)	6.43±0.27	4.82±0.57	0.0005

Rats treated with CCl_4_ (1 ml/kg) were sacrificed at 3 or 24 h after injection. The serum ALT levels and hepatic MDA concentration were measured at 24 h and the hepatic GSH content at 3 h after treatment. Data are expressed as the means ± standard deviation. Statistical analysis was performed with unpaired Student’s t-tests. CCl_4_, carbon tetrachloride; control, vehicle-treated control rats; CCl_4_, CCl_4_-treated rats; ALT, alanine aminotransferase; MDA, malondialdehyde; GSH, glutathione; FW, fresh weight.
